# Protocol for a systematic review and meta-analysis of interventions aimed at delabeling low-risk penicillin allergies with consideration for sex and gender

**DOI:** 10.1186/s13643-024-02671-5

**Published:** 2024-10-14

**Authors:** Mira Maximos, Sameer Elsayed, Colleen Maxwell, Sherilyn K. D. Houle, Ryan Pelletier, Brie McConnell, Andrew Pylypiak, John-Michael Gamble

**Affiliations:** 1 School of Pharmacy, University of Waterloo, Kitchener, ON N2G 2C5 Canada; 2 Women’s College Hospital, Toronto, ON Canada; 3 Departments of Medicine and Pathology and Laboratory Medicine and Epidemiology and Biostatistics, Schulich School of Medicine and Dentistry, London, ON Canada; 4 Woodstock General Hospital, Woodstock, ON Canada

**Keywords:** Antibiotic, Delabeling, Hypersensitivity, Oral challenge, Beta-lactam, Penicillin, Amoxicillin, Allergy, Testing

## Abstract

**Background:**

Approximately, 10% of people report a penicillin allergy; however, more than 90% can safely undergo delabeling after a detailed history, oral challenge, or other investigations such as penicillin skin testing (PST). Although PST is the gold standard, the results can be heterogeneous, and awaiting specialist assessment may take an inordinate amount of time. Therefore, oral provocation challenge has become acceptable for individuals with low-risk penicillin allergy histories. There also appears to be an association with increased prevalence of adverse drug reaction reporting in female individuals, which may translate to penicillin allergy prevalence; however, the evidence has not been assessed through a sex and gender lens. This systematic review will identify and synthesize the findings from studies that report measures of effectiveness and safety of interventions aimed at delabeling penicillin allergies in low-risk individuals. Information related to sex and gender will be extracted, where available, to understand potential differences in allergy reporting and patient outcomes.

**Methods:**

The *Cochrane Handbook for Systematic Reviews of Interventions* and the Centre for Review and Dissemination’s Guidance for Undertaking Reviews in Health Care will be used as frameworks for conducting this systematic review. The literature search will be conducted by a medical librarian (B. M. M.) and will consist of a search strategy to identify and retrieve published studies that meet our inclusion criteria. Studies that require penicillin skin testing (PST) as a step prior to other interventions will be excluded. Integrated knowledge translation involving co-design was carried out for this systematic review protocol creation. Data extraction will be conducted at four levels: (1) study level, (2) patient level, (3) intervention level, and (4) outcome level. A narrative descriptive synthesis of results and risk of bias of all included studies will be provided, and, if relevant, a meta-analysis will be performed.

**Discussion:**

The dissemination of findings from this knowledge synthesis to various stakeholders is intended to inform on options for evidence-based interventions to aid in delabeling penicillin allergies in individuals with a low risk of experiencing a hypersensitivity reaction. Detailed reporting on the characteristics of delabeling interventions as well as the effectiveness of similar interventions will benefit policy makers considering the implementation of a penicillin allergy delabeling protocol. Additionally, findings from this systematic review will report on the current evidence regarding the role of sex and gender in both the prevalence and outcomes associated with the presence of penicillin allergies.

**Systematic review registration:**

PROSPERO CRD42022336457.

## Background

Antibiotics in the beta-lactam family (penicillin, amoxicillin, etc.) are often first-line therapeutic options for many infectious diseases, including skin and soft tissue infections, diabetic foot infections [1–3], respiratory tract infections, bloodstream infections, and other life-threatening conditions [4, 5]. However, approximately 10% of the general population carries the label of a penicillin allergy [5], and up to 15% of hospitalized patients are reported to have a documented penicillin allergy [6].

Applying the label of “penicillin allergy” without sufficient investigation and verification has led to substantial shifts away from the use of the most studied and effective therapeutic regimens towards the use of alternative antimicrobial agents. Compared to beta-lactams, in which penicillins are classified, these agents are associated with higher rates of hospital acquired infections such as methicillin-resistant *Staphylococcus aureus* (MRSA), *Clostridioides difficile*, and vancomycin-resistant *Enterococcus* (VRE) infections [5]. Further, the use of second-line or broad-spectrum non-beta-lactam therapies has been associated with increased hospital length of stay, increased cost of hospitalization, decreased likelihood of infection resolution, infection recurrence, and increased risk of drug toxicity [5].

Following assessment of a purported penicillin allergy, 90 to 95% of individuals have been found to tolerate penicillins [7]. Penicillin allergy delabeling can be carried out through skin testing, serum-specific immunoglobulin E testing for beta-lactams, oral challenge, and delabeling on clinical history [5]. Penicillin skin testing (PST) has historically been the gold standard method of penicillin allergy assessment with a positive predictive value of between 50 to 75%, [5] although interpretation depends on the true prevalence of penicillin allergy in a population. A systematic review and meta-analysis of the diagnostic accuracy of penicillin allergy testing using a variety of modalities was conducted, with data collected from studies involving adult and pediatric cases [8]; for skin tests, sensitivity was low (30.7%, 95% CI of 18.9 to 45.9%) and specificity high (96.8%, 95% CI of 94.2 to 98.3%). Considering the heterogeneity of results for sensitivity, specificity, and predictive value, PST may be a poor screening tool in patients with low-risk penicillin allergies. Given these limitations, oral provocation challenge has become an accepted gold standard method to address penicillin allergy labels in low-risk individuals [5, 7, 9].

For this review, low-risk reactions include mild cutaneous reactions that occurred at least 5 years ago, reactions that are often considered as side effects (e.g., diarrhea or nausea) and reactions of unknown history with tolerance to a within-class beta-lactam in the past [7, 9]. It is important to note that studies vary by the categorization and definition of “low risk.” The importance of this variability became clear during an Ontario Pharmacy Evidence Network (OPEN) Citizen’s Council discussion where feedback from the public was sought on questions related to our study design with a focus on barriers and facilitators for co-design of our protocol. The participants expressed the importance of clear definitions that included patient friendly language for words such as “allergy,” “intolerance,” and “high-risk” and “low-risk” allergies. Based on this feedback, our team will focus on obtaining a list of inclusion criteria from published literature that report penicillin allergy delabeling in “low-risk” populations to determine the most common components of a “low-risk” definition for both direct delabeling and oral challenge.

Another important consideration is the association between penicillin allergy prevalence and outcomes between male and female individuals as well as the associated gender-related variables that may impact these factors, such as healthcare-seeking behaviors. Historically, female sex has been associated with higher rates of adverse drug reactions (ADRs) [10], and the reported prevalence of penicillin allergy may be higher in female adult patients [11–13]. However, it is not clear whether the association is with sex or gender or a combination thereof. Biologic sex has been shown to influence pharmacokinetics, pharmacodynamic processes, and response to drug activity [14]. There is also speculation that the female propensity to drug allergy may be related to a multitude of factors including social constructs such as different utilizations of healthcare [15, 16]. Therefore, there may be both biologic and gender-related factors that may contribute to varied reporting of penicillin allergies and patient outcomes by sex.

The purpose of this systematic review and meta-analysis will include identifying, assessing the quality of, and synthesizing results from studies reporting on the effectiveness and safety of interventions designed to delabel penicillin allergies in the adult population by history, questionnaire, or oral challenge in comparison to no intervention, penicillin allergy skin testing, or other interventions. Sex and Gender-Based Analysis Plus (SGBA +) information will be extracted, where available, to understand sex and gender differences in allergy reporting and outcomes associated with delabeling interventions in the published literature focusing on low-risk allergies.

## Methods and design

The *Cochrane Handbook for Systematic Reviews of Interventions* [17] and the Centre for Review and Dissemination’s Guidance for Undertaking Reviews in Health Care [18] will be used as frameworks for conducting this systematic review and meta-analysis. The PRISMA-P 2015 checklist [19] will be used to include recommended items for this systematic review protocol. COVIDENCE software will be used for study screening. Microsoft Excel will be used for data extraction. The PRISMA-P 2015 checklist will be used to display the steps of the systematic review (Appendix A). The study is registered with PROSPERO (CRD42022336457).

The research questions we aim to answer are as follows: (1) What is the proportion of successfully delabeled penicillin allergies in low-risk patients with a penicillin allergy when delabeling of the allergy is done by history, structured questionnaire, or oral challenge in both in-patient and outpatient settings? and (2) Among published studies, are there sex or gender differences in the population included or outcomes of the interventions?

The primary objective of this systematic review and meta-analysis will be to quantify the percentage of successfully delabeled penicillin allergies in the studied population by proposed intervention, where successful delabeling is defined as an allergy label being removed from a patient’s medical profile as defined by the study authors after successful implementation of an intervention. The secondary objectives will include the following: (1) determining differences between prescribed therapies in intervention and control arms, such as antibiotics used (targeted or broad spectrum, number of antibiotics); (2) duration of antibiotic therapy in intervention and control arms; (3) secondary infections in intervention and control arms; (4) treatment success, reinfection, or readmission in intervention and control arms; (5) cost of therapy or cost savings in intervention and control arms, and (6) prescribing practice changes over time in intervention arm. Sex- and Gender-Based Analysis Plus (SGBA +) information will be extracted, where available to understand sex and gender differences in allergy reporting and outcomes associated with delabeling interventions.

### Eligibility criteria

#### Inclusion

- Studies involving patients with reported penicillin allergies
- Studies that include direct delabeling, delabeling using patient history, or oral challenge
- Randomized controlled trials, non-randomized studies including quasi-experimental studies (e.g., interrupted time series, before and after analyses), cohort studies, case–control studies, and cross-sectional studies
- Primary outcome must include penicillin allergy delabeling
- Adult (18 years of age or older) population
- Studies from all countries will be considered

#### Exclusion

- Non-English language text (due to lack of funding for translator)
- Studies focusing solely on pediatric populations (< 18 years of age) or where data cannot be separated for pediatric and adult individuals
- Animal research and in vitro studies
- Case reports, case series, editorials, opinions, and commentaries
- Delabeling of non-penicillin beta-lactams only
- Studies where all participants underwent penicillin skin testing as a first step

An integrated knowledge translation co-design strategy [20] with involvement of key stakeholders such as pharmacists, physicians, nurses, a public citizens’ council, and administrative as well as leadership personnel will be implemented to optimize the study protocol design. The key stakeholders are involved in the three phases of this knowledge synthesis project: (1) protocol design, (2) critical review of the manuscript, and (3) dissemination of the findings to local practice.

### Search strategy

Prior to embarking on this knowledge synthesis, a search of different electronic databases was conducted based on the Centre for Reviews and Dissemination guidance [18]. This search was performed on February 23, 2022, to ensure that duplication of already existing work is not conducted. The following databases were searched from inception and without limiters for language: (1) The Database of Abstracts of Reviews of Effects (DARE), (2) the Cochrane Database of Systematic Reviews (CDSR), and (3) the International Prospective Register of Systematic Reviews (PROSPERO). One of the studies from the PROSPERO database [21] contained a systematic review protocol focusing on delabeling penicillin allergy by history and/or oral challenge as a secondary outcome; however, the authors of that protocol aim to evaluate the existing published evidence on the effectiveness of penicillin allergy testing and delabeling, with a focus on oral challenge alone without integrating a sex or gender perspective. The full search strategy and results of our protocol can be found in Appendix B. Based on the search of existing or ongoing reviews from the DARE, CDSR, and PROSPERO databases, we were able to determine that a knowledge synthesis on our study question of interest was reasonable to conduct.

### Literature search

A systematic search of the literature was conducted in March of 2022 by a research librarian (BMM) at the University of Waterloo. The search included electronic databases, namely PubMed, the Database of Abstracts of Reviews and Effects (DARE), ClinicalTrials.gov, the Cochrane Database of Systematic Reviews (CDSR) and Library, International Pharmacy Abstracts, medRxiv, Ovid MEDLINE, and Ovid Embase.

The search strategy was developed using a combination of keywords: *penicillin*, or *β-lactams*, and *allergy*, *and direct challenge* and *de-labelling* within an adult population. The keyword strategy was then combined with PubMed’s clinical queries filters, and the full search strategy for PubMed is shown in Appendix C. Any additional database-specific translations of the search strategy are available upon request.

There were no language or date filters applied to the search strategy. In addition, to ensure coverage of related research, all the references from selected publications were reviewed, and all relevant articles were included.

From March 17, 2020, to April of 2023, the research librarian continued to monitor the results feeds of active database search strategies and alerted the researchers of any additional results. The search update was run again in full on October 2, 2023.

The initial literature search will be broad, with a focus on title and abstract screening of the retrieved studies conducted independently by two reviewers after calibration of the first 20 articles screened. The second screen of full-text articles will be performed independently by two reviewers, and the Cohen’s kappa for inter-rater reliability will be reported. Any conflicts between the two reviewers will be resolved through discussion and agreement or, in the case of non-resolution, by involving another reviewer to serve as an adjudicator.

#### Appraising risk of bias

The Cochrane’s risk-of-bias assessment tool [22] will be used to interpret risk of bias in randomized trials, and the Risk of Bias in Non-randomized Studies of Interventions (ROBINS-I) [23] tool will be used as a guide for non-randomized trials and quasi-experimental studies. To reduce risk of bias in study assessment, two reviewers will independently appraise the risk of bias for each study and discuss results to resolve discrepancies. R-Studio or Microsoft Excel will be used, where applicable, to generate interpretable outputs of the assessed risk of bias. Qualitative research components will be narratively described.

#### Data extraction

Data will be extracted using a Microsoft Excel template and reported at 4 levels: (1) study level, (2) patient level, (3) intervention level, and (4) outcome level (as outlined below) and subdivided by data types into the following categories, where possible: (1) descriptive, (2) dichotomous/categorical, and (3) continuous. The data extraction levels, and subdivided categories, can be found in Table 1. Data available in manuscripts and supplementary material will be included; however, authors will not be contacted to obtain any further data.

**Table 1:** Data extraction levels and categories

**Study level**	
Descriptive	Study title, first author, study design, setting, funding sources, required checkbox “does not require penicillin skin testing prior to other forms of delabeling,” includes “low-risk” participants as defined by study authors, include definition of “low risk,” publication status (published or unpublished), aim of study, noted protocol deviations, study limitations
Dichotomous/categorical	Country of study conduct, single or multi-center
Continuous	Year of publication, sample size
**Patient level**	
Descriptive	Patient characteristics (population, inclusion, and exclusion criteria), demographics (sex, age, ethnicity), details regarding initial beta-lactam allergic reaction
Dichotomous/categorical	Type of reported reaction at baseline leading to penicillin allergy label, age at time of reaction, years prior to presentation that reaction occurred
Continuous	Number of participants with reported penicillin allergy, number of participants with penicillin allergy label in each arm at baseline, and number of participants who completed study intervention
**Intervention level**	
Descriptive	Provider who delivered the intervention (allergist/immunologist, pharmacist, nurse, primary care practitioner (physician or nurse practitioner), hospitalist, specialist, patient self-completed, other)
Dichotomous/categorical	Type of intervention (history/questionnaire, oral challenge, skin testing)
Continuous	Not applicable
**Outcome level**	
Descriptive	Class of antibiotics used, antibiotic appropriateness, type of reaction and treatment (if reaction occurred) with delabeling, prescriber practice changes over time
Dichotomous/categorical	Cost of therapy and cost-effectiveness secondary to implemented intervention, rates of reinfection, rates of readmission, rates of *C. difficile* infection, rates of multidrug-resistant organism (MDRO), continuity of delabeling over time, length of hospital stay, inhospital mortality and post-discharge mortality, readmission rates with infective diagnosis
Continuous	Percentage of successfully delabeled penicillin allergies, duration of intravenous antibiotic therapy, total number of antibiotics used

Definitions provided by authors such as “low-risk” penicillin allergy and those for each level and category below will be narratively described and thematically analyzed, where possible. Age will be categorized as mean, median, and range. Variables from PROGRESS elements (race/ethnicity/culture, language, occupation, gender/sex, religion, education, socioeconomic status, and social capital) will be extracted to identify factors that may impact health equity from a gender lens [24]. Reporting of outcome as intention to treat, per protocol, or not specified, and sex/gender analyses, where performed, will be narratively summarized. Outcome measures associated with hypersensitivity reaction will be categorized by reaction type. Where possible, timing of reaction will be categorized as greater than 10 years ago or less than and equal to 10 years ago where defined. If longitudinal follow-up is provided, information regarding antimicrobial use, duration, length of stay, and the presence or absence of MDROs will be collected.

Initial calibration of data extraction will be performed on five full-text articles considered for inclusion by two members of the research team, and conflicts will be resolved by discussion or involvement of a third reviewer. Recording of individuals performing data extraction and the date of data extraction will be maintained in Covidence [25]. If study outcomes differ from protocol to final report, this will be discussed narratively in the systematic review results.

#### Data synthesis and analysis

The intervention effect will be expressed numerically for each study using mean differences for continuous outcomes and relative risks or risk differences for categorical outcomes. For continuous outcomes whereby the measurement scale is not the same across studies, the standardized mean difference, or Cohen’s effect, will be used. The results of single-arm quasi-experimental studies will be reported using mean change for continuous outcomes and proportions for dichotomous outcome.

A meta-analysis will be performed for the primary and secondary outcomes, stratified by intervention type (history/questionnaire, oral challenge, skin testing [reference group for pair-wise meta-analysis]), if two or more studies with consistent patient populations, interventions, and outcomes are captured among included studies. A random-effects meta-analysis using the Knapp-Hartung approach [26] for pair-wise comparison to attain the average intervention effect of penicillin allergy delabeling effectiveness and safety by the proposed intervention will be performed. For quasi-experimental studies that do not have a control arm, a random-effects proportional meta-analysis will be conducted [27].

Heterogeneity of results across studies will be assessed using the chi−square statistic and quantified using the *I*^2^ statistic. If heterogeneity based on *I*^2^ is found to be 75%, the study level effect measures will be reported without a pooled estimate on the forest plot [28]. Pre-defined subgroup analysis will be conducted [29] to explore heterogeneity. The subgroups will include patient sex, provider type (allergist/immunologist, pharmacist, nurse, primary care practitioner (physician or nurse practitioner), hospitalist, specialist, patient self-completed, other), and patient age (less than 65 years versus greater than or equal to 65 years of age) for the primary objective of successful delabeling as well as all secondary objectives expressed in the “outcome level” of the data extraction section will be conducted. If *I*^2^ continues to be above 75%, a meta-analysis will not be conducted, and results will be summarized narratively.

To detect the presence of publication bias, funnel plots for each outcome and, where applicable, statistical methods such as selection models, examining the association between study size and estimated treatment effect, and meta-regression will be adopted, where indicated [30].

## Discussion

Studies of patients with low-risk penicillin allergies have demonstrated the effectiveness and safety of delabeling using history and oral challenge without the use of PST [7, 31–33]. This evidence was culminated in a pooled analysis from a systematic review including evidence from 13 studies with a total sample size of 1202 (range 7–328) showing that across all studies, 3.41% of those receiving a direct oral challenge experienced mild immediate or delayed reactions, there were no serious adverse reactions, and 96.5% of patients could be delabelled [34].

However, much of the literature does not report penicillin allergy testing outcomes by sex, and there is no unifying definition of “low risk” to describe the commonly used term. Therefore, it is unclear whether there is a sex- and/or gender-related difference in success of delabeling, continuity of delabeling, experienced adverse events, and longitudinal outcomes such as future antibiotic use and percentage of patients relabeled in future healthcare interactions. Further, the body of evidence on penicillin allergy risk stratification and delabeling has increased since the publication of the review by Cooper and Colleagues in 2021 [34].

The proposed project will provide a thorough review of the literature on the topic of penicillin allergy delabeling by history and oral challenge while considering sex- and gender-related predictors and outcomes, where described. Feedback from the project steering committee and from the OPEN Citizens’ Council improved the objectives and intended data to be abstracted. The implementation of this co-designed approach into a clinical setting may reduce unnecessary secondary antimicrobial use, reduce adverse outcomes such as secondary infections, and empower patients and care providers with broadening the number and classes of antimicrobials that can be prescribed.

## Data Availability

The datasets during and/or analyzed during the current study are available from the corresponding author on reasonable request.

## Appendix

Appendix A. PRISMA-P 2015 checklist

Appendix B. Review of databases

A search of the DARE database in “all text” search box with the keywords “penicillin” AND “allergy label” in all fields separately and yielded no results returned. To Expand the search, the keywords “penicillin” and “allergy” were searched instead and returned 13 results. Of these, 1 focus on treatment of respiratory tract infections in the pediatric population [35], 1 on pneumonia in a population with cancer [36] and 1 on antimicrobial therapy in a population with cirrhosis [37], 5 are pharmacoeconomic comparisons or cost-effectiveness analyses of therapeutic alternatives or durations of treatment [38–42], 1 study focused on a decision analysis process to determine importance of penicillin skin testing in patients with history of hypersensitivity to penicillin in the setting *of staphylococcus aureus* endocarditis [43], 1 study focused on elective penicillin skin testing prior to amoxicillin oral challenge [44], 1 focuses on efficacy of prophylactic antimicrobials prior to surgery [45], and 1 focused on antimicrobial prophylaxis of Lyme disease [46].

A search of the CSDR database in “all text” advanced search box with the keywords “penicillin” AND “allergy label” returned 22 results. Of these results, 13 were focused on antimicrobial use for treatment of various infectious diseases [47–59], 5 focused on antimicrobial prophylaxis for various medical procedures [53, 60–63], and the 4 remaining studies are not related to antimicrobial therapies or our study question [64–67].

A search of the PROSPERO database with the keywords “penicillin allergy label” returned 6 results of which 1 systematic review and meta-analysis asks a similar question of evaluating the published evidence on effectiveness of penicillin allergy testing and delabeling with the primary objective of reporting prevalence of penicillin allergy as determined by skin testing and/ or direct provocation challenge and one of the secondary outcomes being successful removal of penicillin allergy label [21], 1 protocol focuses on association between penicillin allergy testing in pediatrics and changes in health outcomes [68], 1 protocol focuses on harmful outcomes associated with a penicillin allergy label [69], 1 protocol focuses on involvement of non-allergist specialists in penicillin allergy delabeling [70], 1 focused on cost-effectiveness of penicillin allergy delabeling [71] and the last focuses on diagnostic accuracy of decision support tools in comparison to skin testing followed by oral provocation challenge as the gold standard [72].

Appendix C. PubMed search strategy

PUBMED

MARCH 30, 2022

Search: (((((("Penicillins"[nm]) OR (β-lactam*[Title/Abstract] OR "beta lactam"[Title/Abstract] OR penicillin*[Title/Abstract])) OR ("Penicillins/adverse effects"[Mesh])) AND (allerg*[Title/Abstract] OR hypersensitiv*[Title/Abstract])) AND (((direct[Title/Abstract] OR oral[Title/Abstract]) AND (challenge*[Title/Abstract] OR provocation[Title/Abstract] OR provoked[Title/Abstract])) OR (de-label*[Title/Abstract] OR label*[Title/Abstract] OR delabel*[Title/Abstract]))) NOT (children[Title] OR paediatric*[Title] OR pediatric*[Title])) AND ((((((clinical[Title/Abstract] AND trial[Title/Abstract]) OR clinical trials as topic[MeSH Terms] OR clinical trial[Publication Type] OR random*[Title/Abstract] OR random allocation[MeSH Terms] OR therapeutic use[MeSH Subheading])) OR ((prognos*[Title/Abstract] OR (first[Title/Abstract] AND episode[Title/Abstract]) OR cohort[Title/Abstract]))) OR (((relative[Title/Abstract] AND risk*[Title/Abstract]) OR (relative risk[Text Word]) OR risks[Text Word] OR cohort studies[MeSH:noexp] OR (cohort[Title/Abstract] AND study[Title/Abstract]) OR (cohort[Title/Abstract] AND studies[Title/Abstract])))) OR ((((systematic review[ti] OR systematic literature review[ti] OR systematic scoping review[ti] OR systematic narrative review[ti] OR systematic qualitative review[ti] OR systematic evidence review[ti] OR systematic quantitative review[ti] OR systematic meta-review[ti] OR systematic critical review[ti] OR systematic mixed studies review[ti] OR systematic mapping review[ti] OR systematic cochrane review[ti] OR systematic search and review[ti] OR systematic integrative review[ti] OR meta-analysis OR "meta analysis" OR "meta regress*" OR meta-regress*) NOT comment[pt] NOT (protocol[ti] OR protocols[ti])) NOT MEDLINE [subset]) OR (Cochrane Database Syst Rev[ta] AND review[pt]) OR systematic review[pt]))

Results: 279

Search Update: June 13, 2023 [last search March 23 2022; search date range 2022 – present] Results: 95

Search update: October 2, 2023 [2022 – 2024] Results: 123

## Abbreviations

PST: Penicillin skin testing
MDRO: Multidrug-resistant organism
DARE: The Database of Abstracts of Reviews of Effects
CDSR: The Cochrane Database of Systematic Reviews
PROSPERO: International Prospective Register of Systematic Reviews
MRSA: Methicillin-resistant *Staphylococcus aureus*
*C. difficile*: *Clostridioides difficile*
VRE: Vancomycin-resistant *Enterococcus*
